# AutoFACT: An Automatic Functional Annotation and Classification Tool

**DOI:** 10.1186/1471-2105-6-151

**Published:** 2005-06-16

**Authors:** Liisa B Koski, Michael W Gray, B Franz Lang, Gertraud Burger

**Affiliations:** 1 Robert-Cedergren Center for Bioinformatics and Genomics, Université de Montréal, Montréal, Quebec, Canada; 2 Department of Biochemistry and Molecular Biology, Dalhousie University, Halifax, Nova Scotia, Canada

## Abstract

**Background:**

Assignment of function to new molecular sequence data is an essential step in genomics projects. The usual process involves similarity searches of a given sequence against one or more databases, an arduous process for large datasets.

**Results:**

We present AutoFACT, a fully automated and customizable annotation tool that assigns biologically informative functions to a sequence. Key features of this tool are that it (1) analyzes nucleotide and protein sequence data; (2) determines the most informative functional description by combining multiple BLAST reports from several user-selected databases; (3) assigns putative metabolic pathways, functional classes, enzyme classes, GeneOntology terms and locus names; and (4) generates output in HTML, text and GFF formats for the user's convenience. We have compared AutoFACT to four well-established annotation pipelines. The error rate of functional annotation is estimated to be only between 1–2%. Comparison of AutoFACT to the traditional top-BLAST-hit annotation method shows that our procedure increases the number of functionally informative annotations by approximately 50%.

**Conclusion:**

AutoFACT will serve as a useful annotation tool for smaller sequencing groups lacking dedicated bioinformatics staff. It is implemented in PERL and runs on LINUX/UNIX platforms. AutoFACT is available at .

## Background

Automatic functional annotation is essential for high-throughput sequencing projects. Typically, large datasets undergo annotation by means of "annotation jamborees", where groups of experts are assigned to manually annotate a designated portion of an organism's genome. More recently, various tools have become available to streamline this process [1-9]. However, limitations encountered with these tools are that many require web-submission of data [2], need substantial manual intervention [1,4], supply only a single output format, are part of a large sequence analysis package [3] and most importantly, do not combine a broad range of information resources. To address these shortcomings, we developed a new annotation pipeline, which we term "AutoFACT".

Unique to AutoFACT, is its hierarchal filtering system for determining the most informative functional annotation. This paper describes AutoFACT's functional assignment capabilities, outlining the procedure for annotating unknown nucleotide or protein sequence data. We assess the validity of AutoFACT by comparing annotations to four previously annotated and phylogenetically diverse organisms, including human, yeast and both eukaryotic and bacterial pathogens. AutoFACT has been applied to the EST sequencing project of *Acanthamoeba castellanii*, a free-living soil amoeba and opportunistic human pathogen. This example highlights AutoFACT's performance, which yields a ~50% increase in functional annotations over a top-BLAST-hit approach against NCBI's non-redundant database or against UniProt's expert-annotated UniRef90 database.

## Implementation

AutoFACT is a command-line-driven program written in PERL for LINUX/UNIX operating systems. It uses BioPerl [10] modules to parse and analyze BLAST [11] reports. Average annotation time is 2.5 hours for 5000 sequences of approximately 500 bp in length on a desktop workstation (BLAST time not included). A web version of AutoFACT is available where users can submit up to 10 sequences at a time for annotation. For large sequencing projects, it is recommended that the user download and install the local version of AutoFACT.

## Results

### Methodology

**Figure 1:**
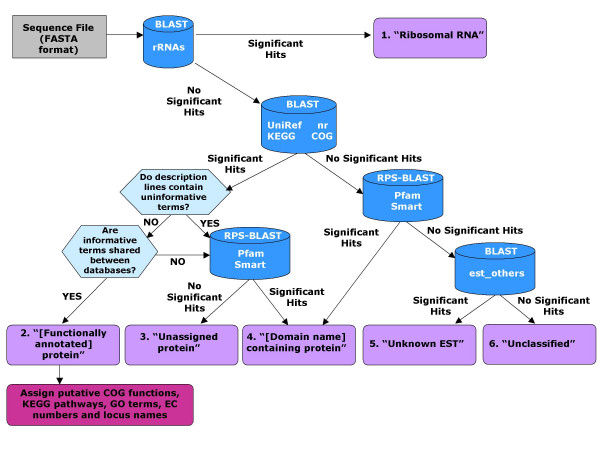
AutoFACT methodology. Sequences are classified into one of six annotation categories (purple boxes). The user decides which bit score cutoff to use (default 40) before a BLAST hit is considered significant. For database references, see text.

AutoFACT takes a single FASTA-formatted sequence file as input, automatically recognizes the sequence type as nucleotide or protein and proceeds to ask the user for preferences regarding which databases to use, the order of database importance and bit score cutoff. The bit score is a measure of sequence similarity independent of the size of the database used (unlike E-values). It is derived from the raw alignment score in which the statistical properties of the scoring system used have been taken into account. Bit scores are normalized with respect to the scoring system and hence can be used to compare alignment scores from different searches [12]. Each sequence in the FASTA-formatted file is then assigned to one of six annotation classes: (1) Ribosomal RNA (rRNA), (2) [Functionally annotated] protein, (3) Unassigned protein, (4) [Domain name]-containing protein, (5) Unknown EST (when using EST data) or (6) Unclassified (Table 1, Figure 1).

**Table 1:** AutoFACT annotation classes

**Annotation Class**	**Hit to LSU or SSU rRNA database**	**Hit to UniRef, nr, KEGG and/or COG**	**Hit is inform-ative**	**Hits share common inform-ative terms**	**Hit to Pfam or Smart**	**Hit to est_others**
**"Ribosomal RNA"**	YES	N/A	N/A	N/A	N/A	N/A
**" [Functionally Annotated] protein"**	NO	YES	YES	YES	N/A	N/A
**"Unassigned protein"**	NO	YES	YES/NO	NO	NO	N/A
**" [Domain name]-containing protein"**	NO	YES/NO	NO	NO	YES	N/A
**"Unknown EST"**	NO	NO	N/A	N/A	NO	YES
**"Unclassified"**	NO	NO	N/A	N/A	NO	NO

AutoFACT assigns classification information, based on a hierarchal system, from a collection of specialized resources, currently nine databases (Table 2), using BLAST comparison [13]. Since not all descriptions from top BLAST hits are genuinely informative, AutoFACT adopts the "uninformative rule" [5], by which the highest scoring BLAST hit with a biologically informative description is considered informative.

**Table 2:** Databases searched and classification information assigned by AutoFACT

**Database**	**Classification Information**	**Reference**
European Ribosomal Database	Large subunit (LSU) ribosomal RNAs Small subunit (SSU) ribosomal RNAs	[25]
Uniprot's UniRef 90	GeneOntology terms Enzyme Commission numbers Locus names	[16,26]
Uniprot's UniRef100		
Clusters of Orthologous Groups (COG)	Functional categories	[27,28]
Kyoto Encyclopedia of Genes and Genomes (KEGG)	Metabolic pathways Enzyme Commission numbers Locus names	[29,30]
Protein Familes Database (Pfam)	Protein domains	[31]
Smart	Signaling domains Domain architectures	[32]
NCBI's non-redundant database (nr)	N/A	[33]
NCBI's est_others database		

Figure 1 outlines the AutoFACT methodology. When analyzing nucleotide data, AutoFACT begins by using BLAST to search the nucleotide sequences in the input file against the set of user-specified databases. If a match to the rRNA dataset is found with a minimum match length and percent sequence identity (default: 50 bp and 84% identity), the sequence is classified as a "ribosomal RNA". If no match is found the sequence is then searched against the remaining set of user-specified databases. In step 2 (or step 1 for protein data), description lines of significant hits, based on a user-specified bit score cutoff (default <40), are examined for the presence of functionally uninformative terms such as 'hypothetical', 'unknown', 'chromosome', etc. When a hit contains an uninformative term, the next best hit is scrutinized and so forth, until a description line without uninformative terms is found, e.g. 'proton-transporting ATP synthase'. The user specifies the number of top BLAST hits the program should filter. In step 3, a search for common terms among the informative hits from each database is performed. For annotation transfer, the user specifies a database order of importance so that informative terms from the first database are searched against informative terms from the remaining databases in a given order. For example, if the user specifies the database order as UniRef90, nr, KEGG and COG, informative terms in the informative hit from UniRef90 are first searched for matches to the informative hits from the other databases. If a match is found between at least one informative term from the UniRef90 hit and at least one other informative database hit (e.g., 'proton-transporting ATP synthase' matches 'H^+^-pumping ATP synthase'), the description line of the UniRef90 hit is assigned to the input sequence. If there are no matches to UniRef90 terms, the informative terms from the informative hit of the next database (nr, in this example) are then queried in the same way as above, until a functionally informative description line has been assigned to the sequence.

We prefer to use UniRef90 as the first database in the order of importance for two reasons. First, as a member of UniProt it is one of the better annotated and curated of the available databases. Second, because UniProt entries with 90% sequence similarity are combined into a single record, the description lines are species-independent and tend to be more general in their descriptions. On the other hand, description lines from NCBI's nr database are often several lines long and contain repetitive information. Testing showed that using various database combinations does not significantly change the annotation results. A user's choice of db order is therefore dependent on the format of the description line one would prefer to assign to the sequence in question (Table 3).

**Table 3:** Database description line formats from ACL00000101 BLAST hits

**Database**	**Description Line**
UniRef90	ATP synthase beta chain related cluster
UniRef100	ATP synthase subunit beta [Salmonella typhimurium]
NCBI's nr	ATP synthase beta chain [Erwinia carotovora subsp. atroseptica SCRI1043] emb|CAG77407.1| ATP synthase beta chain [Erwinia carotovora subsp. atroseptica SCRI1043]
KEGG	atpD; membrane-bound ATP synthase, F1 sector, beta-subunit [EC:3.6.3.14] [KO:K02112]
COG	[C] COG0055 F0F1-type ATP synthase, beta subunit

AutoFACT proceeds to step 4 when there are no common informative terms between any of the databases, or when only uninformative hits are found. In this step, a sequence with significant similarity to one or more sequences in the Pfam or SMART databases is classified as a ' [domain name]-containing' protein or a 'multi-domain-containing protein'. A sequence containing no domains is simply classified as an 'unassigned protein'.

A sequence is also classified as a ' [domain name]-containing protein' when the only significant hit is to a domain database. It is considered 'unclassified' when no hits are found to any of the specified databases. When EST sequences are being annotated, the last step in the annotation pipeline is to check the sequence against NCBI's est_others database. If a significant match is found, the sequence is classified as an 'unknown EST'; otherwise it remains 'unclassified'.

In step 5, functionally annotated sequences are then classified according to KEGG pathways, COG functional groups, Enzyme Commission (EC) numbers, GeneOntology (GO) terms and locus names. Putative KEGG pathways are assigned if an informative term from the automatically assigned description line matches a term in the informative KEGG hit. The same reasoning is used to assign putative COG functional categories. EC numbers [14] are assigned in one of two ways, either from parsing the KEGG description line or by mapping the accession number of the informative UniRef hit to an enzyme *via *ExPASy's enzyme.dat file [15]. GO terms are assigned by mapping the UniRef accession number of the informative hit *via *the gene_association.goa_uniprot file [16].

**Figure 2:**
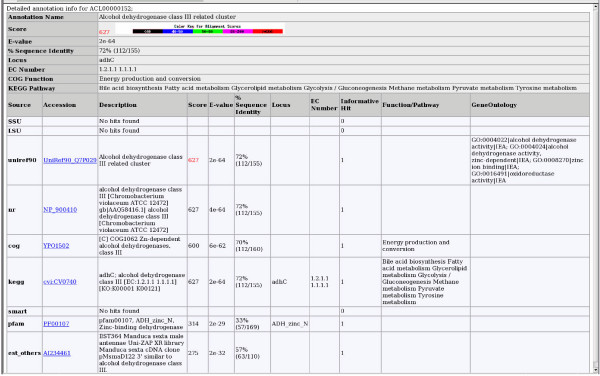
Sample HTML output for AutoFACT annotation of *Acanthamoeba castellanii *EST cluster ACL00000152. Automatic annotation results are displayed at the top of the page and all data used to infer the annotation are represented in the bottom part of the table. Percent sequence identity is the extent to which two (nucleotide or amino acid) sequences, in a High Scoring Segment Pair (HSP), are invariant. In the case of the est_others data, the reported % sequence identity refers to a "translated nucleotide – translated nucleotide" comparison. The "Informative Hit" value specifies whether the first, second, etc., BLAST hit in the corresponding database was informative. The "Color Key for Alignment Scores" displayed at the top of the diagram is from NCBI's BLAST Results page. The scores for the annotation and for the source of the annotation, 627 in this example, are highlighted according to the color key. The page also contains links to relevant database entries.

Three different output formats are generated by AutoFACT: HTML web pages (Figure 2) for easy viewing and browsing, a General Feature Format (GFF) file [17] to facilitate data transfer to the user's private database and a simple tab-delimited text file for easy data extraction and manipulation. A log file is also generated to document all decision-making steps in the annotation process.

**Figure 4:**
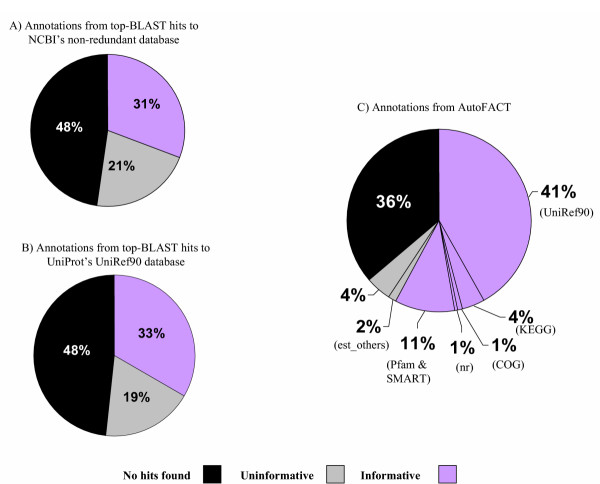
Distribution of informative versus uninformative annotations. *A. castellanii *ESTs (5,130 clusters) were annotated in three ways: (A) by top BLAST hit to NCBI's nr database; (B) by top BLAST hit to UniProt's UniRef90 database; and (C) by AutoFACT. The "uninformative rule" (Andrade *et al*., 1999) was used to query description lines assigned by all methods. AutoFACT yields an ~50% increase in informative annotations compared to top BLAST hits against NCBI's nr and the UniRef90 databases. AutoFACT's annotation source is shown in parentheses ().

### Validation

**Figure 3:**
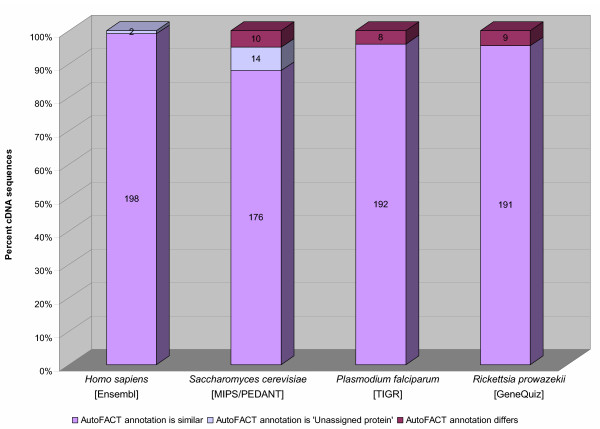
Comparison of AutoFACT annotations across four phylogenetically diverse organisms previously annotated by well-established automatic pipelines. Two hundred previously annotated cDNAs from *Homo sapiens *[Ensembl Annotation Pipeline], *Saccharomyces cerevisiae *[MIPS/PEDANT], *Plasmodium falciparum *[TIGR] and *Rickettsia prowazekii *[GeneQuiz] were re-annotated with AutoFACT using a bit score cutoff of 40 and a database order of importance as follows: UniRef90, KEGG, COG, NCBI's nr, Pfam and SMART. The top 10 BLAST hits to each database were filtered for functionally uninformative terms. BLAST hits to the species itself were considered uninformative. The portion of the bar representing different results from AutoFACT (dark purple) should not be construed as false positives. For example in the case of GeneQuiz (4.5% differences), it is the AutoFACT annotation that is the better of the two in almost all instances (see Results section). Numbers printed directly on columns represent the number of cDNA sequences (out of 200) in each category.

To assess the validity of AutoFACT annotations, we compared results for 200 randomly chosen cDNA sequences across four previously annotated and phylogenetically diverse organisms: i) *Homo sapiens*, annotated by the Ensembl Annotation Pipeline [8]; ii) *Saccharomyces cerevisiae*, annotated by MIPS/PEDANT [18,19]; iii) *Plasmodium falciparum*, annotated by The Institute For Genomic Research (TIGR) [20]; and iv) *Rickettsia prowazekii*, previously annotated by GeneQuiz [5]. We used AutoFACT's default values and considered hits to genes from the same species as uninformative. Figure 3 compares the annotation results of 200 randomly chosen sequences for each species/pipeline.

### *Homo sapiens *[Ensembl]

Comparison of Human Ensembl annotations to AutoFACT revealed no significant differences in annotation assignments. There were 2/200 (1%) sequences that AutoFACT annotated as 'unassigned protein', either because the only BLAST hits were to other human sequences or because the informative terms could not be matched across database sources. Had we been less strict in our annotation criteria and considered hits to the same species as informative, AutoFACT would then have assigned the same annotations as Ensembl to these two sequences. The high similarity between annotation results is primarily due to the fact that the source of most of the Ensembl annotations is UniProt/SWISSPROT, which AutoFACT also uses *via *UniRef90, the database of highest importance in the AutoFACT database order.

### *Saccharomyces cerevisiae *[MIPS/PEDANT]

AutoFACT and PEDANT annotations for a set of 200 cDNAs differed by 5% (10/200). We examined the original annotations for these 10 sequences in the expertly curated Saccharomyces Genome Database (SGD). Because AutoFACT considered hits to *Saccharomyces cerevisiae *as 'uninformative', 6/10 sequences were classified as ' [domain name]-containing proteins'. We do not consider these annotations to be false positives, merely less specific annotations. In 1/10 of the assignments, AutoFACT was better than PEDANT (Table 4). The remaining 3/10 annotations are considered to be false positives, suggesting an overall error rate of 1.5% (3/200).

**Table 4:** Differences found between AutoFACT and PEDANT annotations for *Saccharomyces cerevisiae*

ID	PEDANT Annotation	AutoFACT Annotation	AutoFACT Score	AutoFACT E-value	AutoFACT % Identity
yal048c	vacuolar aspartic protease	**GON1; possible rho-like GTPase involved in secretory vesicle transport**	1724	0.0	50% (360/718)
yhr064c	**SSZ1 – regulator protein involved in pleiotropic drug resistance**	multi-domain protein	651	3.00E-68	28% (154/539)
yhr046c	**INM1 – inositol-1(or 4)-monophosphatase**	*Protein qutG related cluster	378	4.00E-35	31% (99/310)
yhr143w	**DSE2 – glucan 1,3-beta-glucosidase activity**	multi-domain protein	229	2.00E-19	25% (70/278)
yhl043w	**ECM34 – involved in cell wall biogenesis and architecture**	DUP domain-containing protein	205	5.00E-17	36% (26/72)
yal047c	**SPC72 – Stu2p Interactant**	*Repeat organellar protein related cluster	160	2.00E-09	20% (124/620)
yhr167w	**THP2 – subunit of the THO complex, which appears to functionally connect transcription elongation with mitotic recombination**	*Myosin heavy chain related cluster	129	2.00E-06	24% (51/210)
yhr154w	**RTT107 – Establishes Silent Chromatin**	BRCT domain-containing protein	118	4.00E-06	28% (24/83)
yhl020c	**OPI1 – negative regulator of phospholipid biosynthesis pathway**	multi-domain protein	114	5.00E-06	24% (30/123)
yhr196w	**UTP9 – U3 snoRNP protein**	Borrelia_orfA domain-containing protein	104	1.00E-04	19% (75/376)

Annotations in bold are the same as the original annotations found in the Saccharomyces Genome Database. AutoFACT annotations marked with an asterisk (*) are considered false positives.

### *Plasmodium falciparum *[TIGR]

We compared TIGR's preliminary annotations for a set of 200 *Plasmodium falciparum *cDNAs to annotations generated by AutoFACT. TIGR's preliminary annotations are automatically assigned by searching nucleotide and protein databases for "good" matches. At this preliminary stage, none of the annotations are examined or verified by human annotators. We found that between the two fully automatic pipelines, 4% (8/200) of the annotations differed, half of which were annotated by AutoFACT as ' [domain name]-containing proteins' (Table 5). Because TIGR's preliminary annotations have not been examined by human annotators, we cannot estimate the % false positives in this instance.

**Table 5:** Differences found between AutoFACT and TIGR preliminary annotations for *Plasmodium falciparum*

ID	TIGR Preliminary Annotation	AutoFACT Annotation	AutoFACT Score	AutoFACT E-value	AutoFACT % Identity
1396.m03572	PF14_0675 reticulocyte binding protein 2 homolog B, putative Reticulocyte Binding protein;	multi-domain protein	157	1E-10	18% (60/320)
1396.m03591	PF14_0655 RNA helicase-1, putative	Eukaryotic translation initiation factor 4A related cluster	1591	1E-177	79% (310/388)
1396.m03721	PF14_0530 ferlin, putative	heat shock protein DNAJ pfj4	534	6E-53	40% (103/252)
1396.m04144	PF14_0112 POM1, putative	Twinkle related cluster	152	6E-08	38% (34/89)
1396.m04178	PF14_0078 HAP protein	Asp domain-containing protein	535	8E-55	26% (100/371)
1396.m04220	PF14_0036 acid phosphatase, putative	Metallophos domain-containing protein	134	2E-08	20% (45/220)
1396.m04244	PF14_0015 aminopeptidase, putative	hydrolase, alpha/beta fold family	179	5E-12	22% (66/288)
1396.m04296	PF14_0382 metalloendopeptidase, putative	multi-domain protein	118	0.000006	16% (50/297)

### *Rickettsia prowazekii *[GeneQuiz]

AutoFACT annotations for *Rickettsia prowazekii *[21] were compared to annotations previously assigned by GeneQuiz ([5,22]). AutoFACT differed from GeneQuiz annotations at 4.5% (9/200) of the sequences, yet differed only by 1% (2/200) from the more accurate original annotations [21], which are based on human inspection and include phylogenetic information. GeneQuiz estimates an overall error rate of 2.5–5%, which is confirmed in our comparison here (Table 6). Based on these automatic annotation results, AutoFACT is the more accurate of the two pipelines, with an error rate of only 1%.

**Table 6:** Differences found between AutoFACT and GeneQuiz annotations for *Rickettsia prowazekii*

ID	Gene Quiz Annotation	AutoFACT Annotation	AutoFACT Score	AutoFACT E-value	AutoFACT % Identity
RP103	PKM101 CONJUGATION PROTEINS (TRAL), (TRAM), (TRAA), (TRAB), (TRAC), (TRAB), (TRAC), (TRAD), (TRAN), (TRAE), (TRAO), (TRAF), (TRAG), ENTRY EXCLUSION PROTEIN (EEX), (KIKA), (KORB), (KORA) AND ENDONUCLEASE (NUC) GENES, COMPLETE CDS (TRAM) (TRAB) (TRAB) (TRA	**VIRB4 PROTEIN related cluster**	4159	0.0	100% (805/805)
RP151	NEMPA PROTEIN PRECURSOR.	**Aspartyl/glutamyl-tRNA(Asn/Gln) amidotransferase subunit B related cluster**	2004	0.0	82% (398/483)
RP259	D-STEREOSPECIFIC PEPTIDE HYDROLASE PRECURSOR.	Penicillin binding protein 4* related cluster	2048	0.0	96% (398/414)
RP268	NADH-UBIQUINONE OXIDOREDUCTASE CHAIN 2 (EC 1.6.5.3).	**Heme exporter protein B related cluster**	794	3E-84	74% (160/215)
RP282	**NADH DEHYDROGENASE SUBUNIT 2.**	*HyfB domain-containing protein related cluster	1821	0.0	74% (380/512)
RP287	CAVEOLIN-2.	**VIRB8 PROTEIN related cluster**	1047	1E-114	85% (212/247)
RP291	CONJUGAL TRANSFER PROTEIN TRBI.	**VIRB10 PROTEIN related cluster**	2016	0.0	85% (413/483)
RP293	CONJUGAL TRANSFER PROTEIN TRAG.	**VIRD4 PROTEIN related cluster**	3002	0.0	97% (577/591)
RP414	LPS BIOSYNTHESIS RFBU RELATED PROTEIN.	*Glycosyltransferase related cluster	1614	1E-180	92% (314/338)

Annotations in bold are the same as the original annotations by Andersson *et al*. (1998).

AutoFACT annotations marked with an asterisk (*) are considered false positives.

### Case Study: *Acanthamoeba castellanii*

AutoFACT is currently used by the Protist EST Program (PEP) [23], a pan-Canadian genomics initiative involving investigators at six Canadian universities. The objective of PEP is to survey, through EST sequencing, the expressed portions of the genomes of a phylogenetically comprehensive selection of protists (30–40 of these mostly unicellular eukaryotes).

Under the PEP initiative, 12,937 individual EST reads yielding 5,130 clusters (consensus sequences) have been obtained to date for *A. castellanii*. We compared AutoFACT annotations for these clusters to annotations taken from top BLASTx hits against NCBI's nr database and from top BLASTx hits against UniProt's well-annotated UniRef90 database. AutoFACT compared the *A. castellanii *sequences against a total of seven databases. UniRef90, KEGG, COG and NCBI's nr were searched using BLASTx ; Pfam and SMART were searched using RPS-BLAST; and NCBI's est_others database was searched using tBLASTx. In each instance, a bit score cutoff of 40 was used and the top 10 BLAST hits were filtered for uninformative terms. The database order of importance was UniRef90, KEGG, COG, NCBI's nr. Figure 4 shows an ~50% increase in functionally informative annotations with AutoFACT (58% informative hits) compared to the quick and easy top-BLAST-hit approach (~ 32%). Scanning the top 10 hits for informative terms in AutoFACT's UniRef90 source alone results in a 10% increase in informative annotations over the top-BLAST-hit approach against both nr and UniRef90. This result demonstrates the power of the "uninformative rule" alone. As such there is a significant decrease (from 19% to 6%) in 'uninformative' hits when using AutoFACT. By searching against the domain databases Pfam and SMART, AutoFACT reduces the number of 'no hits found' by approximately 10% in comparison to the datasets annotated by the top-BLAST-hit approach.

AutoFACT annotations for each organism mentioned above can be viewed at 

## Conclusion

To efficiently and fully exploit the wealth of sequence data currently available, thorough and informative functional annotations are paramount. Considering the ever-growing number of EST sequencing projects, it becomes increasingly important to fully automate the annotation process and to make optimal use of the various available annotation resources and databases. Because no two annotation systems are exactly alike, choice of system is very much dependent on the user's end goal.

AutoFACT uses a hierarchal filtering system for determining the most informative functional annotation. It provides a means of classification by identifying EC numbers, KEGG pathways, COG functional classes and GeneOntology terms. AutoFACT supplies three different output formats and a log file, which are versatile and adaptable to user requirements. Importantly, it allows users to maintain data locally, whereas many other systems require sequence submission elsewhere for annotation. By combining multiple resources, AutoFACT associates sequences with a broad range of biological classifications and has proven to be very powerful for annotating both EST and protein sequence data. The *A. castellanii *case study shows that in comparison to the 'quick and easy' top-BLAST-hit approach against either NCBI's nr or UniProt's UniRef databases, AutoFACT substantially improves functional annotations of sequence data. Comparisons to other well-established annotation pipelines show that AutoFACT performs equally well and in some cases better than the alternative. We have also demonstrated that AutoFACT exhibits an equivalent level of performance (1–2% error rate) when it is used to annotate sequences across different domains of life.

Finally, we caution that over-prediction is common when using sequence similarity to infer protein function. Examples of similar sequences that do not share the same or even related functions have been documented [24]. Automatic annotations therefore may require further validation in certain cases.

## Availability and requirements

Project name: AutoFACT

Project homepage: 

Operating system(s): LINUX/UNIX

Programming language: PERL

Other requirements: BioPerl and BLAST

License: GNU General Public License (GPL)

Any restrictions to use by non-academics: None

## Authors' contributions

LBK designed, developed and implemented AutoFACT. MWG provided the *Acanthamoeba castellanii *data used to test and validate AutoFACT. GB and BFL supervised the study, making significant design contributions. All authors read and approved the final manuscript.
